# Acceptability of patient-centered, multi-disciplinary medication therapy management recommendations: results from the INCREASE randomized study

**DOI:** 10.1186/s12877-023-03876-4

**Published:** 2023-03-10

**Authors:** Noah I. Smith, Ashley I. Martinez, Mark Huffmyer, Lynne Eckmann, Rosmy George, Erin L. Abner, Gregory A. Jicha, Daniela C. Moga

**Affiliations:** 1 University of Kentucky College of Pharmacy, Lexington, KY USA; 2 Harvard Pilgrim Health Care Institute and Harvard Medical School, Boston, MA USA; 3 University of Kentucky Healthcare, Lexington, KY USA; 4 LLC Consulting Services, Lexington, KY PRO2RX USA; 5 Sanders-Brown Center On Aging, University of Kentucky, Lexington, KY USA; 6 University of Kentucky College of Public Health, Lexington, KY USA; 7 University of Kentucky College of Medicine, 789 S Limestone, Lexington, KY 40536 USA

**Keywords:** Deprescribing, Polypharmacy, Medication therapy management, Pharmacotherapy, Clinical trials

## Abstract

**Background:**

Polypharmacy and inappropriate medications may be a modifiable risk factor for Alzheimer’s Disease and Related Dementias (ADRD). Medication therapy management (MTM) interventions may mitigate medication-induced cognitive dysfunction and delay onset of symptomatic impairment. The objective of the current study is to describe an MTM protocol for a patient-centered team intervention (pharmacist and non-pharmacist clinician) in a randomized controlled trial (RCT) directed at delaying the symptomatic onset of ADRD.

**Methods:**

Community dwelling adults 65 + years, non-demented, using ≥ 1 potentially inappropriate medications (PIM) were enrolled in an RCT to evaluate the effect of an MTM intervention on improving medication appropriateness and cognition (NCT02849639). The MTM intervention involved a three-step process: (1) pharmacist identified potential medication-related problems (MRPs) and made initial recommendations for prescribed and over-the-counter medications, vitamins, and supplements; (2) study team reviewed all initial recommendations together with the participants, allowing for revisions prior to the finalized recommendations; (3) participant responses to final recommendations were recorded. Here, we describe initial recommendations, changes during team engagement, and participant responses to final recommendations.

**Results:**

Among the 90 participants, a mean 6.7 ± 3.6 MRPs per participant were reported. Of the 259 initial MTM recommendations made for the treatment group participants (N = 46), 40% percent underwent revisions in the second step. Participants reported willingness to adopt 46% of final recommendations and expressed need for additional primary care input in response to 38% of final recommendations. Willingness to adopt final recommendations was highest when therapeutic switches were offered and/or with anticholinergic medications.

**Conclusion:**

The evaluation of modifications to MTM recommendations demonstrated that pharmacists’ initial MTM recommendations often changed following the participation in the multidisciplinary decision-making process that incorporated patient preferences. The team was encouraged to see a correlation between engaging patients and a positive overall response towards participant acceptance of final MTM recommendations.

**Trial registration:**

Study registration number: clinicaltrial.gov NCT02849639 registered on 29/07/2016.

**Supplementary Information:**

The online version contains supplementary material available at 10.1186/s12877-023-03876-4.

## Background

Many prior studies have provided evidence that medication therapy management (MTM) can lead to improved health and economic outcomes [1–4]. MTM involves five core components: availability of a personal medication record, medication therapy review, development of a medication-related action plan, intervention and/or referral, and documentation and follow-up of medication changes or lack thereof [5–7]. Though most MTM services share these five basic elements, there is heterogeneity in how these services are operationalized. Specifically, there is variability in how potentially inappropriate medications (PIMs) are identified, whether certain medications are targeted specifically, the types of recommendations made, and patient’s acceptance of the proposed changes from an MTM intervention. Additionally, patient and pharmacist engagement with prescribing clinicians varies, [5–7] though evidence shows that pharmacist-prescriber-clinician teams engaging together in MTM activities results in better medication optimization outcomes [2, 8–10]. It is important to characterize MTM-related services in collaborative practices in order to estimate their impact on patient health outcomes, especially for MTM services targeting vulnerable populations such as older adults receiving PIMs.

We recently completed the INtervention for Cognitive Reserve Enhancement in delaying the onset of Alzheimer's Symptomatic Expression (INCREASE) study, a randomized controlled trial where we tested an MTM intervention that actively involved the patient, a board-certified geriatric pharmacy specialist (BCGP), and a non-pharmacist clinician [11, 12]. INCREASE was designed to evaluate the effect of the MTM intervention on changes in medication appropriateness and cognitive function; study data included comprehensive information on health history, medication use and experience with medication taking, as well as the process of implementing the MTM intervention. We previously reported on the successful implementation of the MTM intervention that translated into an improved medication appropriateness at the one-year follow-up [12]. The current study characterizes the stepwise process of delivering the MTM intervention in the INCREASE trial with the goal of helping to fill a qualitative gap in the literature surrounding MTM interventions, specifically focused on patient-centered, multidisciplinary approaches. The specific approach described, including details of the process, provides a model for future evidence based, multidisciplinary MTM interventions that may be implemented rationally in practice.

The objectives of the current manuscript are twofold: (1) describe the recommendations made by the study BCGPs using participant-reported medical and medication histories for all INCREASE participants, prior to randomization to either the MTM intervention (specific medication recommendations plus provision of educational materials on inappropriate medication use) or usual care (i.e., only provision of educational materials on inappropriate medication use), and (2) describe final recommendations for patients randomized to the MTM intervention. The second objective describes (a) revisions to the preliminary baseline MTM recommendations over the course of the intervention, and (b) participant response to the MTM recommendations following the intervention.

## Methods

### INCREASE study overview

The INCREASE study was a randomized controlled trial enrolling community-dwelling adults 65 years and older who did not have dementia and were using at least one PIM as defined in the 2015 Beers Criteria (the most recent version at the time of the study) [13]. Complete details of the INCREASE protocol and results are available elsewhere and briefly described below [11, 12]. After 1:1 randomization that was stratified based on baseline amyloid burden, participants randomized to the control group received usual care with educational pamphlets on medication appropriateness for older adults and risks associated with polypharmacy. In addition to educational materials, participants randomized to the MTM intervention met with the BCGP and a non-pharmacist study clinician (e.g., nurse practitioner, neurologist) to discuss the baseline recommendations. This meeting allowed for 1) participant education on risks, benefits, and alternatives to optimize medication use; and 2) the collection of additional relevant information, including participant beliefs, preferences, and treatment goals. During the MTM team meeting, final recommendations were formalized, and the details of any relevant revisions to the baseline recommendation were noted in the pre-specified data collection forms.

The INCREASE study was approved by the University of Kentucky Institutional Review Board (IRB #43239) and all the study participants provided informed consent. The protocol for the study was registered on clinicaltrials.gov (NCT02849639) on 29/07/2016, in accordance with the relevant guidelines and regulations or in accordance with the Declaration of Helsinki. Study data were collected and managed using the Research Electronic Data Capture (REDCap), a secure, web-based software platform designed to support data capture for research studies [14, 15].

### Baseline recommendations (all INCREASE study participants)

Before randomization, comprehensive medication reviews were conducted by BCGPs for all participants using participant-reported medical conditions and information on dose, frequency, indication, duration of treatment, tolerability, and adverse drug reactions for all prescription medications, vitamins, and supplements. The BCGP medication review process involved 1) assessing the clinical appropriateness of each medication using the Beers Criteria [13] and Medication Appropriateness Index (MAI); [16] 2) evaluating potential drug-drug and drug-disease interactions in accord with the above and also taking into account prescription label information; and 3) assessing whether medication regimens followed relevant disease-specific evidence-based guidelines [13, 17, 18]. Of note, blood laboratory work results, electronic medical records, and previous therapies (e.g., medication failures) were not available to BCGPs when devising baseline recommendations, but were available to the clinician member of the MTM team. Following randomization, the MTM recommendations were only shared with those participants randomized to the intervention group (*N* = 46). Recommendations for the control group were recorded in the study database but not shared with those participants.

During the INCREASE study period, the pharmacy team of two BCGPs utilized drug and health information resources (e.g., Lexicomp and UpToDate [Wolters Kluwer Health Inc. Riverwoods, IL]), Beers Criteria [13], relevant guidelines (e.g., Diabetes Standards of Care [17] and Clinical Practice Guidelines for Hypertension [18]), and clinical judgement to justify their recommendations. Each recommendation was reviewed by both BCGPs and a consensus pharmacy recommendation was decided via discussion. Detailed information for each recommendation was then entered into a series of pre-specified study protocol data collection forms, allowing for systematic categorization of recommendations as either: 1) medication discontinuation with or without tapering; 2) switch to a different medication; 3) dose adjustment (e.g., decrease dose, adjust dose for organ function/tolerability, or increase dose); 4) new medication initiation; 5) drug or disease monitoring recommendation (e.g., vital signs, falls risk, sedation); or 6) a non-pharmacologic recommendation (e.g., sleep hygiene, avoiding gastroesophageal reflux triggers, referral for diagnostic workup). Baseline recommendations were also categorized by pharmacologic class and over the counter (OTC) or supplement status of the medication prompting a baseline MTM recommendation. A full schematic for medication categorization is available in the supplementary material (see Supplementary Table S1).

### Final recommendations (MTM intervention group only)

After 1:1 stratified randomization, study pharmacists met with the participant and study clinician to deliver the MTM intervention. During the intervention, the team gathered further information from the patient and discussed baseline recommendations together, in-person, with additional context provided by the participant on their health status, needs, and preferences. Because health status and medication use in participants may have changed in the time between the baseline assessment and the initial MTM recommendation, comparison of baseline to final recommendations was limited to those baseline MTM recommendations that proposed medication changes at the time of the initial MTM study visit. The non-medication related recommendations (see supplementary material for additional information) were discussed during the team MTM intervention, but they were not included in the present analysis.

Participant responses to each final MTM recommendation for participants randomized to the MTM intervention were collected at the conclusion of the initial MTM intervention visit using a standardized form where the participant selected his or her response to the recommendation as 1) willing to change, 2) refusing to change, 3) needing to confer with a primary care provider or other specialist (e.g., cardiologist), or 4) not applicable (e.g., the participant had already discontinued the medication, dose adjustment was no longer warranted per clinical judgement). In this manuscript we are describing in detail the immediate participant response as recorded following the baseline intervention. The impact of the intervention on medication appropriateness is described in detail elsewhere [12].

## Results

### Baseline characteristics

Of the 104 participants screened, 90 were eligible and randomized in the INCREASE study. Of these, 46 participants were randomized to the MTM intervention group. The mean (SD) age at enrollment was 73.9 years (6.0). The majority of the participants reported female gender (64%) and white race (89%), with an average of 16.5 (2.8) years of education. The mean Charlson Comorbidity Index score was 1.9 (1.9), with participants reporting an average of 12.8 (4.8) total medications 2.4 (1.4) medications per participant were identified as PIMs per 2015 Beers Criteria. Supplementary Table S2 provides additional information on baseline characteristics for all the participants in the INCREASE study as well as for those randomized to the MTM intervention.

### Baseline recommendations (all INCREASE study participants) 

A total of 602 pre-randomization recommendations were made across the 90 INCREASE participants, averaging 6.7 ± 3.3 MTM recommendations per participant and ranging from 1 to 17 baseline recommendations per participant (median [IQR] of 7 [4, 8.9]). Table 1 shows the distribution of medication categories associated with baseline recommendations and the types of recommendations provided.

**Table 1:** Baseline MTM recommendations by medication category^†^ and recommendation type among all INCREASE trial participants (*N* = 90)

*Recommendations*		*MTM recommendations (total: N* = *602)*	
		*N*	*%*
*Medication category†*	Cardiometabolic	138	22.9%
	Gastrointestinal	102	16.9%
	Pain management	87	14.5%
	Anticholinergics	77	12.8%
	Vitamins and supplements	76	12.6%
	Neuropsychiatric	67	11.1%
	Other	55	9.1%
*Recommendation type*	Dose adjustment	170	28.2%
	Switch to preferred agent	166	27.6%
	Drug and disease monitoring	101	16.8%
	Non-pharmacologic therapy	77	12.8%
	Discontinuation	45	7.5%
	Initiation of new medication	43	7.1%

^†^See supplementary Table S1 for full listing of medication categories

The most common class of medications with recommendations were cardiometabolic agents (*N* = 138, 23%), followed by medications for gastrointestinal conditions (*N* = 102, 17%), pain management (*N* = 87, 15%), anticholinergics (*N* = 77, 13%), vitamins and supplements (*N* = 76, 13%), neuropsychiatric agents (*N* = 67, 11%), and other medications (*N* = 55, 9%). Across all baseline recommendations, one-third (*N* = 201, 33%) were prompted by use of PIMs available on the US market as over-the counter (OTC) products without a prescription. The most frequent OTC medications included proton pump inhibitors, vitamins/supplements, antihistamines, OTC non-steroidal anti-inflammatories, aspirin, and H_2_ receptor antagonists.

The most common type of baseline recommendation was continuation of therapy with dose adjustment (e.g., decrease pain medication dose, intensify antihypertensive medication dose) (*N* = 170, 28%). Second most common were therapeutic switches to a less risky pharmacotherapeutic alternative (*N* = 166, 28%; e.g., de-escalate from a proton pump inhibitor to a H_2_ receptor antagonist ± calcium-based antacid; switch from a first-generation to non-sedating second-generation antihistamine). Monitoring (*N* = 101, 17%) and non-pharmacologic recommendations (*N* = 76, 13%) accounted for about one-third of all baseline MTM recommendations. The most frequent monitoring recommendations involved recommending objective testing (e.g., blood pressure, blood chemistry/organ function tests) and recording self-reported measures (e.g., dizziness, pain). Non-pharmacologic recommendations most frequently involved counseling for fall prevention strategies with and without physical therapy referral, dietary and lifestyle changes for gastrointestinal conditions, non-pharmacologic pain management, and sleep hygiene.

Although recommendations to discontinue medications were relatively less frequent (*N* = 46, 8%), those medications most commonly associated with a baseline MTM recommendation to discontinue included vitamins/supplements and medications with therapeutic duplication (e.g., participant was taking two separate antihistamines for seasonal allergies). All recommendations for initiation of a new medication (*N* = 43, 7%) involved treating an unmet clinical need and/or initiating a preventative medication, most often a guideline-recommended statin or aspirin in the setting of cardiovascular risk factors.

### Final recommendations (Intervention Group only)

Following randomization, INCREASE participants who were assigned to MTM (*N* = 46) met with the BCGP and a non-pharmacist clinician. There were 296 baseline recommendations across the MTM arm’s participants. Of these, 37 recommendations (12.5%) proposed at baseline did not relate directly to a medication change and were therefore excluded from the final recommendation analysis included in this manuscript. An account of these 37 excluded recommendations is provided in supplementary table S3. Finalized, unblinded MTM recommendations that were directly related to a medication change, comprised 259 of the original 602 blinded baseline recommendations, averaging 5.6 (SD 2.3) MTM recommendations per participant.

The distribution of final recommendations by medication category was as follows: cardiometabolic (*N* = 58, 22%), pain management (*N* = 42, 16%), vitamins and supplements (*N* = 38, 15%), anticholinergics (*N* = 32, 12%), gastrointestinal (*N* = 32, 12%), neuropsychiatric (*N* = 31, 12%), and other (*N* = 26, 10%). The distribution of by recommendation type was as follows: dose adjustment (*N* = 98, 38%), switch to preferred agent (*N* = 92, 36%), drug and disease monitoring (*N* = 30, 12%), discontinuation (*N* = 26, 10%), and initiation of a new medication (*N* = 13, 5%).

Table 2 shows the results of the patient-pharmacist-clinician team MTM interventions after randomization. Less than half of the baseline recommendations were revised through the team discussion and deliberation process (*N* = 104, 40%). Baseline recommendations were least likely to be revised for vitamins/supplements and cardiometabolic medications, or with a recommended dose adjustment or new initiation. Conversely, baseline recommendations were the most likely to be revised when involving GI therapy and pain management medications, or for recommended medication monitoring or discontinuation. The most frequent reasons for revisions were due to missing information relevant to the participant’s medical history (e.g., a missing diagnosis for Barrett’s esophagus warranting proton pump inhibitor use) and/or missing medication information (e.g., previous failure or intolerability of a guideline-preferred pharmacotherapeutic agent).

**Table 2:** Revision status of MTM recommendations through intervention delivery, and participant responses to final MTM recommendations by medication category and recommendation type (*N* = 259 final recommendations) among INCREASE intervention group participants (*N* = 46 participants) ^a^

*Recommendations*		*Recommendation was revised during the MTM intervention*	*Participant response to final MTM recommendation after revision, if applicable*			
			Willing to change (*N* = 118)	Must first confer with another provider* (*N* = 99)	Refusal to change (*N* = 15)	Not applicable** (*N* = 27)
		N (%)	N (%)	N (%)	N (%)	N (%)
*Medication category*	Cardiometabolic (*N* = 58)	19 (33%)	19 (33%)	27 (47%)	1 (2%)	11 (19%)
	Pain management (*N* = 42)	23 (55%)	20 (48%)	15 (36%)	4 (10%)	3 (7%)
	Vitamins and supplements (*N* = 38)	11 (29%)	20 (53%)	12 (32%)	4 (11%)	2 (5%)
	Anticholinergics (*N* = 32)	13 (41%)	19 (59%)	10 (31%)	1 (3%)	2 (6%)
	Gastrointestinal (*N* = 32)	19 (59%)	14 (44%)	15 (47%)	1 (3%)	2 (6%)
	Neuropsychiatric (*N* = 31)	13 (42%)	12 (39%)	16 (52%)	1 (3%)	2 (6%)
	Other (*N* = 26)	6 (23%)	14 (54%)	4 (15%)	3 (12%)	5 (19%)
*Recommendation type*	Dose adjustment (*N* = 98)	36 (37%)	38 (39%)	44 (45%)	4 (4%)	12 (12%)
	Switch to preferred agent (*N* = 92)	45 (49%)	48 (52%)	34 (37%)	3 (3%)	7 (8%)
	Drug and disease monitoring (*N* = 30)	9 (30%)	14 (47%)	8 (27%)	2 (7%)	6 (20%)
	Discontinuation (*N* = 26)	9 (35%)	13 (50%)	8 (31%)	5 (19%)	0 (0%)
	Initiation (*N* = 13)	5 (38%)	5 (38%)	5 (38%)	1 (8%)	2 (15%)

^a^Cell percentages are displayed as a percent of the row total

^*^Another provider could be a primary care physician, specialist such as a cardiologist, or other non-study prescribing clinician

^**^Reasons for lack of applicability included medication use having been appropriately modified since baseline medication use information was collected, or the proposed medication change was no longer clinically relevant given additional information from the participant and/or MTM team discussion

Upon receiving the finalized MTM recommendations, participants responded about half the time that they were willing to make the changes proposed (*N* = 118, 46%), and often needed to confer with a primary care provider or other clinical specialist (*N* = 99, 38%) before making a decision, but rarely refused to make the proposed changes (*N* = 15, 6%). In some cases (*N* = 27, 10%), the recommendation was no longer clinically relevant and participant responses were recorded as not applicable. Lack of applicability arose from medication use having been appropriately modified since baseline medication use information was collected (*N* = 11), or from the proposed medication change no longer being clinically relevant given additional information from the participant and/or MTM team discussion (*N* = 16). A full account of these 27 recommendations in provided in supplementary table S4.

Participant willingness to adopt recommended MTM changes was highest for vitamins/supplements and anticholinergic agents, and for recommendations involving a pharmacotherapeutic switch. Participants most often responded that they needed to confer with a primary care provider or other specialist when the MTM recommendations included psychiatric, GI, and cardiometabolic medications, or for dose adjustments or medication switches. Participant refusal to adopt final recommended changes (*N* = 15, 6%) was low across all medication categories and recommendation types in the INCREASE trial MTM intervention. Refusal was highest among recommendations involving vitamins and supplements (*N* = 4) or pain management (*N* = 4), as well as for recommendations involving medication discontinuation (*N* = 5).

## Discussion

This study describes MTM recommendations for participants enrolled in the INCREASE trial. The most common medication categories flagged at baseline included 1) cardiometabolic medications, 2) gastrointestinal medications, 3) pain management medications, 4) anticholinergics, and 5) vitamins/supplements. The most common types of recommendations made at baseline were 1) dose adjustments and 2) switches to more appropriate therapeutic alternatives.

Notably, BCGP recommendations were not strictly medication related. In this study, many MTM recommendations did not directly involve a medication change, but rather addressed other potential medical problems (e.g., disease monitoring, referral for diagnostic workup or physical therapy, addition of non-pharmacologic therapies). Each of the top five medication categories identified in the analysis for baseline recommendations included at least some OTC medication options, and one-third of baseline MTM recommendations involved a medication available OTC. OTC products are available without a prescription and were identified frequently as PIMs (13% of all baseline recommendations and 15% of final recommendations). Thus, our study points to the importance of educating patients on the risk–benefit profile of OTCs and the role of pharmacists in OTC stewardship.

Among the final MTM recommendations analyzed, 40% underwent revision compared to the baseline MTM recommendation provided. This reflects the potential for several factors to influence recommendations as more information is gathered in a multidisciplinary MTM intervention. Notably, input from the patient on previous therapies, medication tolerability, feasibility/adherence, and condition severity may help inform the MTM team’s final decision-making process. Our results demonstrate that engaging the patient in a team-based intervention may result in patient-motivated revisions to baseline recommendations. This comparison of pre-intervention recommendations to final recommendations after team deliberation has not been discussed in previous literature.

Participant responses indicated willingness to make recommended changes about half of the time and a need to confer with a primary care provider or other clinical specialist about one third of the time. This was interpreted as generally positive, since participants were most often willing to either accept the final recommendation as specific, or to further engage with another healthcare provider to seek additional medical advice. While participant refusal to change was generally low, our findings suggest that patients may be less willing to adopt MTM recommendations for certain categories of medications or for certain recommendation types. Previous literature has addressed acceptability of MTM recommendations [19–23]; however, the recommendation type and medication category have not been described in relation to participant willingness to make changes. Further research is needed to determine if willingness to adhere long-term to recommendations is impacted by the type of recommendation and medication in question.

Though extensive medication and medical histories were collected from participants, the baseline recommendations were limited to self-reported information before randomization, and complete clinically relevant information was not always available to the BCGP at baseline (e.g., renal function from an electronic medical record). This finding indicates that pharmacists engaged in MTM processes need access to relevant clinical information and an opportunity for direct engagement with prescribers and the patient who have first-hand knowledge of such clinical variables. In addition, chart review may not capture all information necessary to make a patient-centered MTM recommendations, which has not been reviewed in previous literature [19–23]. As health status, medication use and tolerability change over time, there is a need to routinely review previous recommendations and adjust them as needed to reflect the patient’s current needs.

There are several limitations to this study. The 2015 Beers Criteria [13]. was used in the study, which was the latest version at the time of the study. During the INCREASE trial, updated Beers Criteria were published by the American Geriatrics Society in 2019 [24]. As an example, the 2015 Beers Criteria recommended caution in aspirin use for primary cardiovascular event prevention among adults aged ≥ 80 years. In contrast, the 2019 Beers Criteria expanded the recommendation to caution in aspirin use for both primary cardiovascular event and colorectal cancer prevention for adults aged ≥ 70 years. This may limit generalizability as medical treatments, guidelines, and prescribing patterns evolve over time in response to scientific evidence. Another limitation of this descriptive study was that the INCREASE participant experiences may not be generalizable to populations that have different distributions of demographic and health characteristics. Similarly, local prescribing practices and the use of PIMs observed in the INCREASE trial may not be representative of the entire US population today. Additionally, the number of study pharmacists and prescribing clinicians was small. Ability for multiple pharmacists to independently review and adjudicate the categorization of MTM recommendations would strengthen future studies. Though this study adds to the body of descriptive literature on baseline MTM trial recommendations, further studies in diverse populations are needed to identify culturally appropriate MTM strategies, as well as to allow a more detailed examination of prescribing inequities that might influence MTM outcomes over time. When teams gather to critically evaluate an individual’s medication use process (i.e., diagnostician/prescriber, dispenser, and medication user), open dialogue may facilitate transparency in strategic medication use decisions. Negotiation of an evidence-based approach to medication use should be to the individual’s unique combination of diseases, medications, clinical status, and very importantly – personal preference that may have its roots in cultural/social/racial/ethnic diversity.

It is important to note that not all MTM interventions are equivalent. The INCREASE trial modeled its intervention on a foundation of multidisciplinary team interaction with active participant engagement. This is often beyond the scope of traditional, community pharmacy-based MTM models in practice today. The present results suggest that medical advice from a patient-centered team with multiple healthcare perspectives is both appealing to patients and may elicit a stronger patient acceptance of MTM recommendations. Further studies characterizing patient responses to different MTM models are needed to determine whether the qualities such as mode of delivery and multidisciplinary involvement impact long-term recommendation adherence, and/or influences patient outcomes.

## Conclusion

Multidisciplinary interventions such as the pharmacist-clinician-patient MTM team used in the INCREASE study may hold promise for improving health-related outcomes among community dwelling persons. Thorough characterization of MTM interventions is needed to specifically describe the nuances of MTM approaches for making recommendations. It is also critical for guiding future endeavors in the area of MTM science. The present data demonstrate that the recommendations suggested by patient-centered multidisciplinary healthcare teams can be dynamic and complex, and that participant responses may vary depending on the medication targeted and the type of recommendation proposed.

## Supplementary Information

**Additional file 1:** **Supplementary Table S1.** Medication categorization schematic for medications prompting baseline medication recommendations in the INCREASE trial. **Supplementary Table S2.** Baseline characteristics of all INCREASE trial participants, and those randomized to the MTM intervention. **Supplementary Table S3.** Full account of baseline recommendations for the MTM intervention arm that were excluded from final recommendation analysis (N=37). **Supplementary Table S4.** Full account of final recommendations designated as not applicable (N=27)

## Data Availability

Data collected will be shared upon reasonable request from corresponding author.

## Abbreviations

AD: Alzheimer’s disease
ADRD: Alzheimer’s disease and related dementias
BCGP: Board-certified geriatric pharmacist
INCREASE: INtervention for cognitive reserve enhancement in delaying the onset of Alzheimer's symptomatic expression
MRP: Medication-related problem
MTM: Medication therapy management
PIM: Potentially inappropriate medication
UK IRB: University of Kentucky Institutional Review Board
