# Supplementary material for: Acceptability of patient-centered, multi-disciplinary medication therapy management recommendations: results from the INCREASE randomized study
Source: BMC Geriatr. 2023 Mar 10;23:137. doi: 10.1186/s12877-023-03876-4 (PMC9999619; doi:10.1186/s12877-023-03876-4)
Supplement: Supplementary file 1 — Additional file 1: Supplementary Table S1. Medication categorization schematic for medications prompting baseline medication recommendations in the INCREASE trial. Supplementary Table S2. Baseline characteristics of all INCREASE trial participants, and those randomized to the MTM intervention. Supplementary Table S3. Full account of baseline recommendations for the MTM intervention arm that were excluded from final recommendation analysis (N=37). Supplementary Table S4. Full account of final recommendations designated as not applicable (N=27) [file 12877_2023_3876_MOESM1_ESM.docx]

Supplementary Table S1: Medication categorization schematic for medications prompting baseline medication recommendations in the INCREASE trial.

| **Broad Drug Category** | **Subcategory** | **Generic Drug Name***** |
| --- | --- | --- |
| Cardiometabolic | Antihypertensive  (excluding ß-blockers) | Amlodipine, benazepril, clonidine, doxazosin, furosemide, hydrochlorothiazide, irbesartan, lisinopril, losartan, terazosin, torsemide, triamterene |
|  | Antihyperlipidemic | Atorvastatin, colesevelam, colestipol, ezetimibe, gemfibrozil, *omega-3 acid ethyl esters,* pravastatin, simvastatin |
|  | Aspirin | *Aspirin* |
|  | Antiarrhythmic | Amiodarone, bisoprolol, carvedilol, metoprolol |
|  | Antidiabetic | Glipizide, insulins, metformin, sitagliptin |
|  | Antiplatelet/anticoagulant  (excluding aspirin) | Apixaban, clopidogrel, rivaroxaban |
| Pain Management  (excluding aspirin) | Non-steroidal anti-inflammatory | Celecoxib, diclofenac, *ibuprofen,* indomethacin, meloxicam, nabumetone, *naproxen* |
|  | Gabapentinoid | Gabapentin, pregabalin |
|  | Opioid | Codeine, hydrocodone, tramadol |
|  | Skeletal muscle relaxant | Baclofen, carisoprodol, cyclobenzaprine, tizanidine |
|  | Acetaminophen | *Acetaminophen* |
| Vitamin/ supplement | Vitamin | *Combination multivitamins and prenatal vitamins, vitamin B3, vitamin B6, vitamin B7, vitamin B9, vitamin B12, vitamin C, vitamin D, vitamin E* |
|  | Mineral | *Calcium, iron, magnesium,* potassium, *selenium* |
|  | Other OTC vitamin/supplement,  or herbal medication | *Alpha lipoic acid, coenzyme Q10, cranberry, glucosamine/chondroitin, inositol, krill oil, red yeast rice, resveratrol, turmeric* |
| Anticholinergic | Antihistamine | *Cetirizine, chlorpheniramine, dimenhydrinate, diphenhydramine, doxylamine, fexofenadine,* hydroxyzine, *levocetirizine, loratadine, meclizine* |
|  | Antimuscarinic | Fesoterodine, oxybutynin, solifenacin, trospium |
| Gastrointestinal | Proton pump inhibitor | Dexlansoprazole, *esomeprazole, lansoprazole, omeprazole pantoprazole, rabeprazole* |
|  | H_2_ receptor antagonist | *Famotidine, ranitidine* |
|  | Other gastrointestinal agent | *Docusate*, promethazine |
| Neuropsychiatric | Selective serotonin reuptake inhibitor | Citalopram, escitalopram, fluoxetine, paroxetine, sertraline |
|  | Benzodiazepine | Alprazolam, clonazepam, temazepam |
|  | Serotonin-norepinephrine reuptake inhibitor | Duloxetine, venlafaxine |
|  | Tricyclic antidepressant | Amitriptyline, doxepin, imipramine, nortriptyline |
|  | Antiepileptic | Carbamazepine, oxcarbazepine, primidone |
|  | Other neuropsychiatric agent | Aripiprazole, bupropion, buspirone, donepezil, lisdexamfetamine, *melatonin,* mirtazapine, pramipexole, vortioxetine, zolpidem |
| Other | Hormonal agent | Anastrozole, conjugated estrogens, estradiol, testosterone, tamsulosin |
|  | Thyroid agent | Levothyroxine |
|  | Bone agent | Alendronate, denosumab |
|  | Respiratory agent | Budesonide/formoterol, fluticasone/vilanterol, *guaifenesin,* umeclidinium |
|  | Nasal medication | Flunisolide, *fluticasone, phenylephrine* |
|  | Infectious disease/ immunomodulatory agent | Adalimumab, doxycycline, hydroxychloroquine, pneumococcal vaccines |

***Italicization of drug names indicate that the drug was categorized as an over-the-counter product. In some cases, OTC status varies by product strength and/or formulation (e.g., ibuprofen, oxybutynin, potassium, vitamin B12), or may have been made available OTC to patients in the United States since the study took place (e.g., diclofenac gel).

Supplementary Table S2: Baseline characteristics of all INCREASE trial participants, and those randomized to the MTM intervention

| **Characteristic** | **INCREASE trial**  **(N=90)** | **MTM intervention**  **(n=46)** |
| --- | --- | --- |
| Baseline age *(years):*  *Mean (SD)*  *Median (IQR)* | 73.9 ± 6.0  72.5 [65, 79] | 73.6 ± 5.6  72.5 [69, 78] |
| Female: n (%) | 58 (64%) | 34 (74%) |
| White race: n (%) | 80 (89%) | 40 (87%) |
| Education *(years completed)*  *Mean (SD)*  *Median (IQR)* | 16.5 ± 2.8  16 [14, 18] | 16.5 ± 2.6  16.5 [15, 18] |
| Charlson Comorbidity Index *(score)*  *Mean (SD)*  *Median (IQR)* | 1.9 ± 1.9  1 [0, 2] | 2.0 ± 2.1  1 [1, 2] |
| Total number of medications  *Mean (SD)*  *Median (IQR)* | 12.8 ± 4.8  13 [9,16] | 12.7 ± 5.0  12 [8, 15] |
| Number of PIMs*  *Mean (SD)*  *Median (IQR)* | 2.4 ± 1.4  2 [1, 3] | 2.5 ± 1.2  3 [1, 3] |

^*^As described in the 2015 Beers Criteria

MTM: Medication Therapy Management; PIM: Potentially Inappropriate Medications

Supplementary Table S3: Full account of baseline recommendations for the MTM intervention arm that were excluded from final recommendation analysis (N=37)

| *Nature of baseline recommendation excluded from final recommendation analysis* | *Number of recommendations*  *(total: N=37)* |
| --- | --- |
| Sleep hygiene counseling | 16 |
| Recommendation for diagnostic or differential diagnostic workup with a healthcare specialist and/or labwork | 11 |
| Non-pharmacologic dietary recommendation (e.g., avoiding trigger foods in gastroesophageal reflux) | 3 |
| Non-pharmacologic pain relief (e.g., for arthritis) | 3 |
| Non-pharmacologic device use (e.g., pessary) | 2 |
| Recommendation for psychiatric counseling and stress management | 1 |
| Recommendation to ensure vaccines up-to-date | 1 |

Supplementary Table S4: Full account of final recommendations designated as not applicable (N=27)

| *Nature of final recommendation with not applicable (N/A) designation* | *Number of recommendations*  *(total: N=27)* |
| --- | --- |
| Medication change proposed would be unsuitable given patient information (e.g., previous failure of preferred therapy, change would pose compliance barrier or intolerable side effects) | 12 |
| Dose adjustment already made | 7 |
| Medication change no longer relevant given MTM discussion (e.g., disease status changes justified PIM use) | 4 |
| Switch to preferred agent already made | 2 |
| Medication already discontinued | 2 |
